# Binary addition in a living cell based on riboregulation

**DOI:** 10.1371/journal.pgen.1007548

**Published:** 2018-07-19

**Authors:** Arantxa Rosado, Teresa Cordero, Guillermo Rodrigo

**Affiliations:** Instituto de Biología Molecular y Celular de Plantas, Consejo Superior de Investigaciones Científicas–Universidad Politécnica de Valencia, Valencia, Spain; Universidad de Sevilla, SPAIN

## Abstract

Synthetic biology aims at (re-)programming living cells like computers to perform new functions for a variety of applications. Initial work rested on transcription factors, but regulatory RNAs have recently gained much attention due to their high programmability. However, functional circuits mainly implemented with regulatory RNAs are quite limited. Here, we report the engineering of a fundamental arithmetic logic unit based on *de novo* riboregulation to sum two bits of information encoded in molecular concentrations. Our designer circuit robustly performs the intended computation in a living cell encoding the result as fluorescence amplitudes. The whole system exploits post-transcriptional control to switch on tightly silenced genes with small RNAs, together with allosteric transcription factors to sense the molecular signals. This important result demonstrates that regulatory RNAs can be key players in synthetic biology, and it paves the way for engineering more complex RNA-based biocomputers using this designer circuit as a building block.

## Author summary

In this work, we have engineered a distinctive genetic system, based on regulatory RNAs that control the process of protein translation, that is able to perform arithmetic logic computations (additions) in a single bacterial cell. The system expresses as output fluorescent proteins according to the molecular concentrations of the inputs (binary code). In the future, this circuitry might be instrumental to develop smart bacterial cells that can make appropriate decisions after certain computation for biomedical applications.

## Introduction

In 1945, von Neumann established the foundations of the logic architectures behind computers in his famous “First draft of a report on the EDVAC” [1]. There, the arithmetic logic unit (ALU) appeared as a principal element in the central processing unit. An ALU is a digital-like circuit that performs arithmetic and logic operations over bits of information. Certainly, today’s computers mount complex ALUs to deal with large volumes of information [2]. But in an emerging scenario of unconventional modes of computation [3], we could wonder whether ALUs, even if in simple forms, are implementable by other means. In particular, is it possible to engineer genetically such a device in a single living cell? Importantly, this question had a positive answer with the engineering of a genetic half adder in mammalian cells [4], by combining transcription factors (TFs) and RNA-binding proteins. A half adder is a basic implementation of an ALU to perform the binary sum of two bits of information. This requires generating two output channels, one for the *sum* (multiple of 1) and another for the *carry* (multiple of 2). Later, a genetic half adder was also engineered in bacterial cells exploiting combinatorial transcriptional regulation [5]. However, these designs are centered on regulatory proteins, which are limited in number, especially those with high propensity for composability and orthogonality with the host machinery, and do not allow an easy computational design of *de novo* sequences. In this regard, and even though ground-breaking work is being accomplished on circuit design automation [6], directed evolution of TFs [7], and *de novo* protein design [8], alternatives to protein-based regulation are required.

In recent years, RNA has been exploited as an ideal substrate to engineer gene expression programs that robustly run *in vivo*, thanks to its functional versatility [9, 10] and model-based designability at the nucleotide level [11, 12]. Examples of this suitability are novel mechanisms of gene expression control through the modulation of transcription with non-coding RNAs [13–15], or chimeric RNA molecules integrating different domains that are able to transduce molecular signals [16–18]. Moreover, efforts in RNA synthetic biology to increase the sophistication of the designer systems have led to combinatorial logic gates [19], serial cascades [20, 21], a feed-forward loop [22], and a pulse counter [23]. In this work, we go one step further with the engineering of a genetic half adder in *Escherichia coli* centered on regulatory RNAs. In particular, we focused on riboregulators of translation initiation [24, 25] to implement our design. The whole system also relies on TF-mediated regulation, especially to sense the molecular signals and express accordingly those riboregulators.

Interestingly, a genetic half adder would allow mounting a common response against two different molecules acting individually (mediated by the sum), and mounting a new response when they act together (mediated by the carry). This would be useful, for instance, in scenarios in which there is synergy between molecules [26].

## Results

A half adder receives two input signals and processes them to generate two output responses. In this work, isopropyl β-D-1-thiogalactopyranoside (IPTG) and anhydrotetracycline (aTc) are the two molecules that work as input signals. Moreover, the expressions of a superfolder green fluorescent protein (sfGFP) [27] and a monomeric red fluorescent protein (mRFP1) [28] constitute the output responses. The computation is accomplished in two different genetic modules, both receiving IPTG and aTc as inputs, but each producing one different output. The first genetic module implements a XOR logic gate and generates the sum in the red fluorescence channel. That is, mRFP1 is expressed in presence of IPTG alone or aTc alone. The second genetic module implements an AND logic gate and generates the carry in the green fluorescence channel. That is, sfGFP is expressed in presence of both IPTG and aTc. To implement these logic circuits, we used a synthetic PL-based promoter repressed by LacI, PLlac, and another PL-based promoter repressed by TetR, PLtet [29]. This way, the genes controlled by these two promoters can be induced by IPTG and aTc, respectively, in a strain constitutively expressing the TFs LacI and TetR (here *E*. *coli* MG1655-Z1).

**Fig 1:**
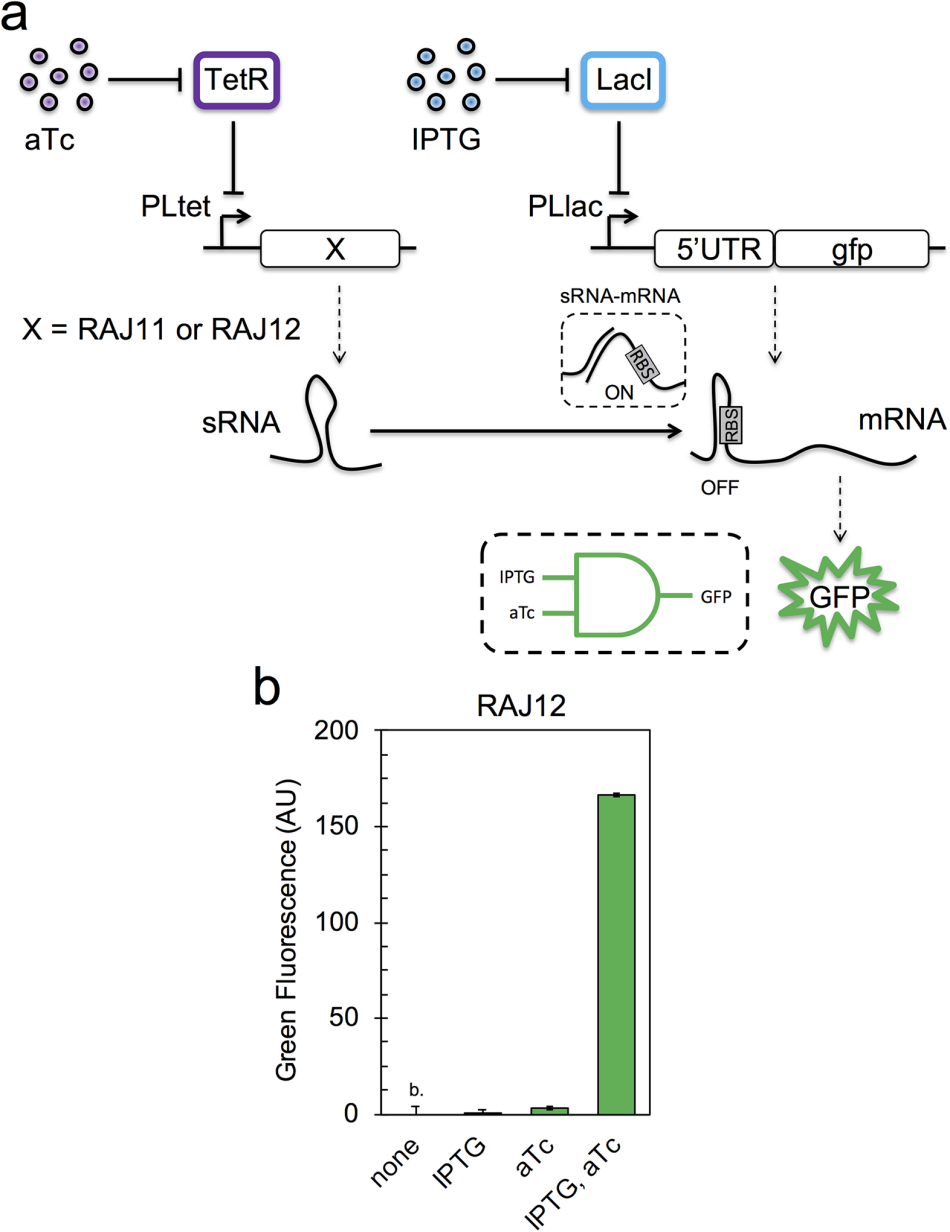
RNA-based AND logic gate. a) Scheme of the gene regulatory circuit. A gene coding for a GFP, initially repressed (OFF state), is activated by a riboregulator (ON state). Solid lines denote regulations, while dashed lines correspond to transcription or translation processes. b) Normalized green fluorescence for each induction condition (IPTG, aTc) when the circuit is implemented with the RAJ12 sRNA and sfGFP. Error bars correspond to standard deviations over three replicates (b. indicates fluorescence below cell autofluorescence). The truth table of AND reads 00|0, 10|0, 01|0, and 11|1.

We started by engineering the AND logic gate, as this circuit is much simpler than the XOR logic gate. The AND behavior was conceived as the expression, on the one hand, of a *cis*-repressed messenger RNA (mRNA) coding for a GFP with the PLlac promoter and, on the other hand, of a small RNA (sRNA) able to *trans*-activate translation with the PLtet promoter (**Fig 1A**); a scheme already proposed [30, 31]. *Cis*-repression can be achieved by trapping the ribosome binding site (RBS) in the stem of a strong hairpin formed in the 5’ untranslated region (5’ UTR) of the mRNA, and *trans*-activation requires a suitable seed region between the sRNA and that hairpin [12]. According to our previous work with the riboregulatory system RAJ11 [31], there is a substantial increase in green fluorescence when both IPTG and aTc are present in the medium, a result obtained again here in new conditions (section A in **S1 Appendix**). In this case, a GFPmut3b [32] was used as output, following the original system. The no apparent expression in the other induction conditions (readouts even below the fluorescence of cells that do not express GFP) indicated a tight RBS repression. In addition, we considered the riboregulatory system RAJ12 [31] to implement another AND logic gate. We also observed in a fluorometer a substantial increase in green fluorescence only with both inducers, now with sfGFP, but apparently with less dynamic range (**Fig 1B**). The tight RBS repression was also noticeable in this case. Indeed, previous single-cell analyses of the systems RAJ11 and RAJ12 (by flow cytometry) revealed fluorescence distributions almost coincident with the distribution coming from cells that do not express GFP, even with plasmids of high copy number [31]. Accordingly, we decided to keep the RAJ12-based AND logic gate (implemented in one single plasmid, pRHA12) as one final module, and exploit the riboregulatory system RAJ11 for the engineering of the XOR logic gate.

**Fig 2:**
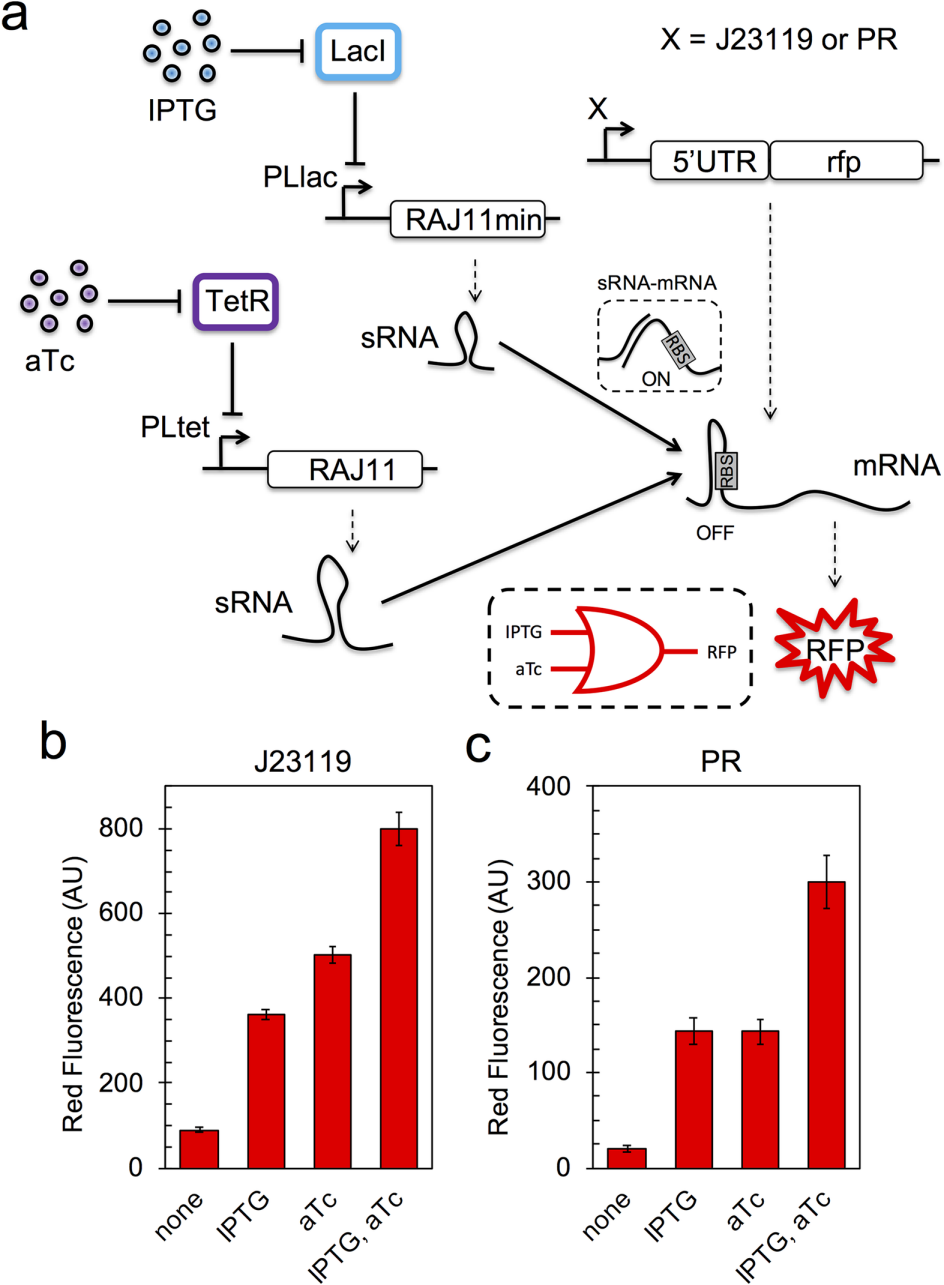
RNA-based OR logic gate. a) Scheme of the gene regulatory circuit. A gene coding for an RFP, initially repressed (OFF state), is activated in parallel by two riboregulators (ON state). Solid lines denote regulations, while dashed lines correspond to transcription or translation processes. b, c) Normalized red fluorescence for each induction condition (IPTG, aTc). Error bars correspond to standard deviations over three replicates. The promoter that controls the RFP expression (mRFP1 in both cases) is J23119 in b) and PR in c). The truth table of OR reads 00|0, 10|1, 01|1, and 11|1.

Our next goal was to engineer an OR logic gate, proposing two *trans*-activations of translation in parallel [19]. For that, we placed a *cis*-repressed mRNA coding for the mRFP1 under the control of a constitutive promoter (J23119 [33]), and the RAJ11 sRNA under the control of the PLtet promoter. Subsequently, we designed a minimal version of such sRNA (RAJ11min), also able to *trans*-activate the translation of that mRNA. This was done to avoid repeated regulatory genes in the circuit, which presumably enhances genetic stability. The RAJ11 and RAJ11min sRNAs produce the same intermolecular base pairs with the corresponding 5’ UTR. The RAJ11min sRNA was then expressed with the PLlac promoter (**Fig 2A**). We found a significant expression boost either with IPTG or aTc (**Fig 2B**). The similar expression levels indicated fully functionality of the RAJ11min sRNA. Moreover, we found that the expression levels are almost the double upon induction with both IPTG or aTc. This is expected if we assume that (synthetic) riboregulation, in contrast to transcriptional regulation, rests on decreased binding affinity *in vivo* (sRNA-mRNA interaction) and then operates in the linear regime, without reaching saturation [34–36]. Afterwards, we decided to replace the promoter that controls mRFP1 expression. In particular, we chose the PR promoter from λ phage [37]. In absence of the TF cI, this promoter is also constitutive in *E*. *coli*. We found a similar expression pattern as before, but with less than half expression levels (**Fig 2C**). This is in tune with previous work on promoter characterization showing that the J23119 promoter is stronger than the PR promoter [33]. Running the EFM calculator, devised for assessing evolutionary failure modes [38], we obtained a RIP score (lower is more stable) of 270.7 for this last OR logic gate (implemented with the RAJ11 and RAJ11min sRNAs and the PR promoter); while it would be 773.0 if this gate were implemented with two copies of RAJ11 or 373.9 with two copies of RAJ11min.

**Fig 3:**
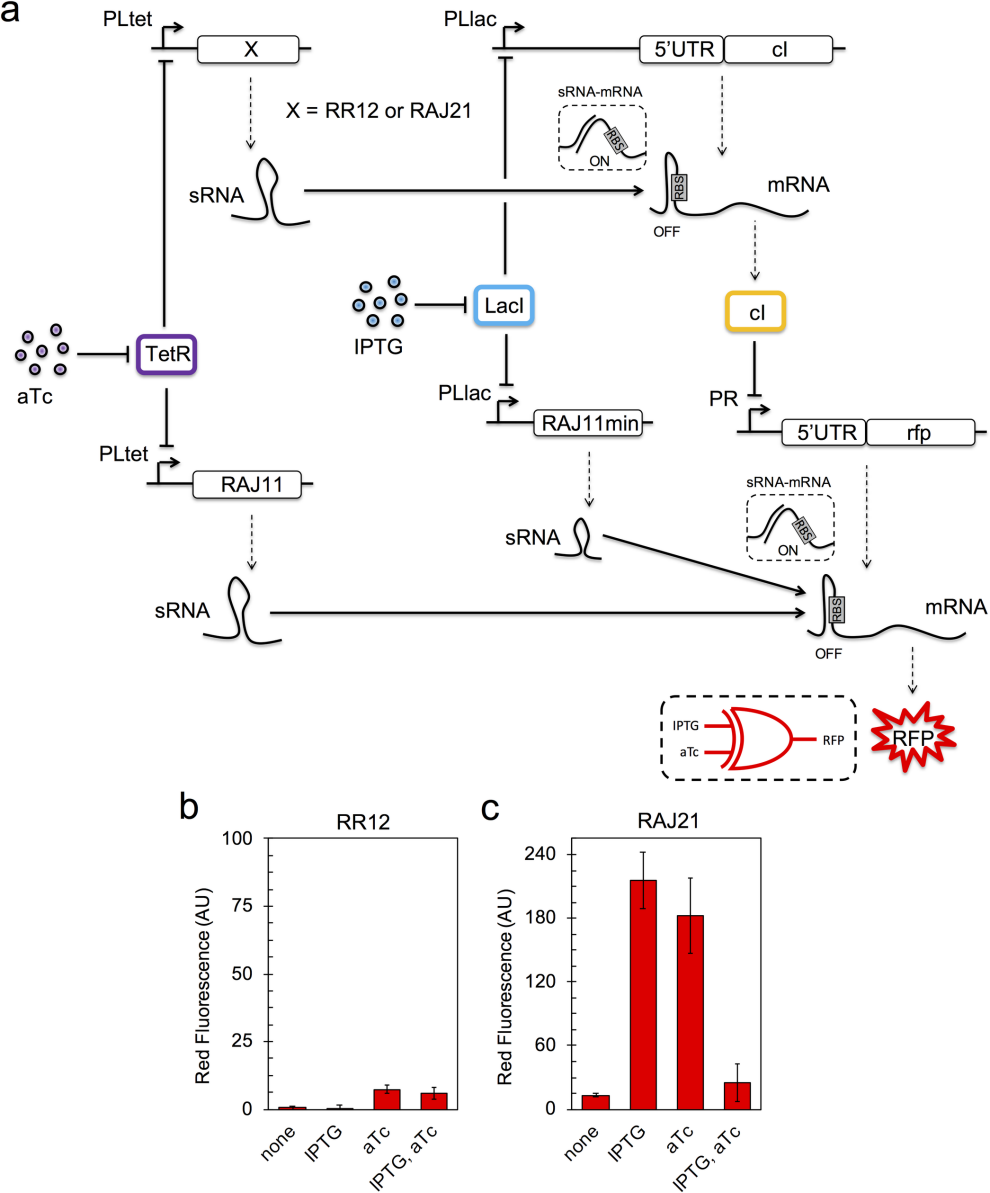
RNA-based XOR logic gate. a) Scheme of the gene regulatory circuit. On top of the OR logic gate, a gene coding for cI, initially repressed, is activated by a riboregulator to switch off RFP (OFF state). Solid lines denote regulations, while dashed lines correspond to transcription or translation processes. b, c) Normalized red fluorescence for each induction condition (IPTG, aTc). Error bars correspond to standard deviations over three replicates. The riboregulator that activates cI is the RR12 sRNA in b) and the RAJ21 sRNA in c). The truth table of XOR reads 00|0, 10|1, 01|1, and 11|0.

In addition, we conceived the XOR behavior as the combination of such an OR logic gate and an additional AND logic gate. To this end, we expressed, on the one hand, a *cis*-repressed mRNA coding for cI with the PLlac promoter and, on the other hand, of a sRNA able to *trans*-activate translation of that gene with the PLtet promoter (**Fig 3A**). This way, cI is only expressed in the presence of both IPTG and aTc (AND behavior). In turn, cI represses the PR promoter, which expresses mRFP1. To implement this system, we tried two different riboregulatory systems, RR12 [24] and RAJ21 [31], knowing that the apparent dynamic range is much larger for RR12. However, we only found the intended behavior with the system RAJ21, as mRFP1 was not expressed with the system RR12 (**Fig 3B and 3C**). We argued that cI was relatively expressed with only IPTG or aTc when the system RR12 implements the logic circuit, and that this cI expression was sufficient to repress the PR promoter. As cI is a potent repressor [39] and the circuit was expressed from a high-copy plasmid, any expression leakage, due to inefficient transcriptional or translational control, can end in repression of mRFP1. In terms of translation, previous single-cell analyses (by flow cytometry) revealed a small expression leakage from the *cis*-repressed mRNA in the case of RR12 [24], but not in the case of RAJ21 [31]. Hence, the RAJ11/RAJ21-based XOR logic gate (implemented in one single plasmid, pRHA40) resulted in the other module.

**Fig 4:**
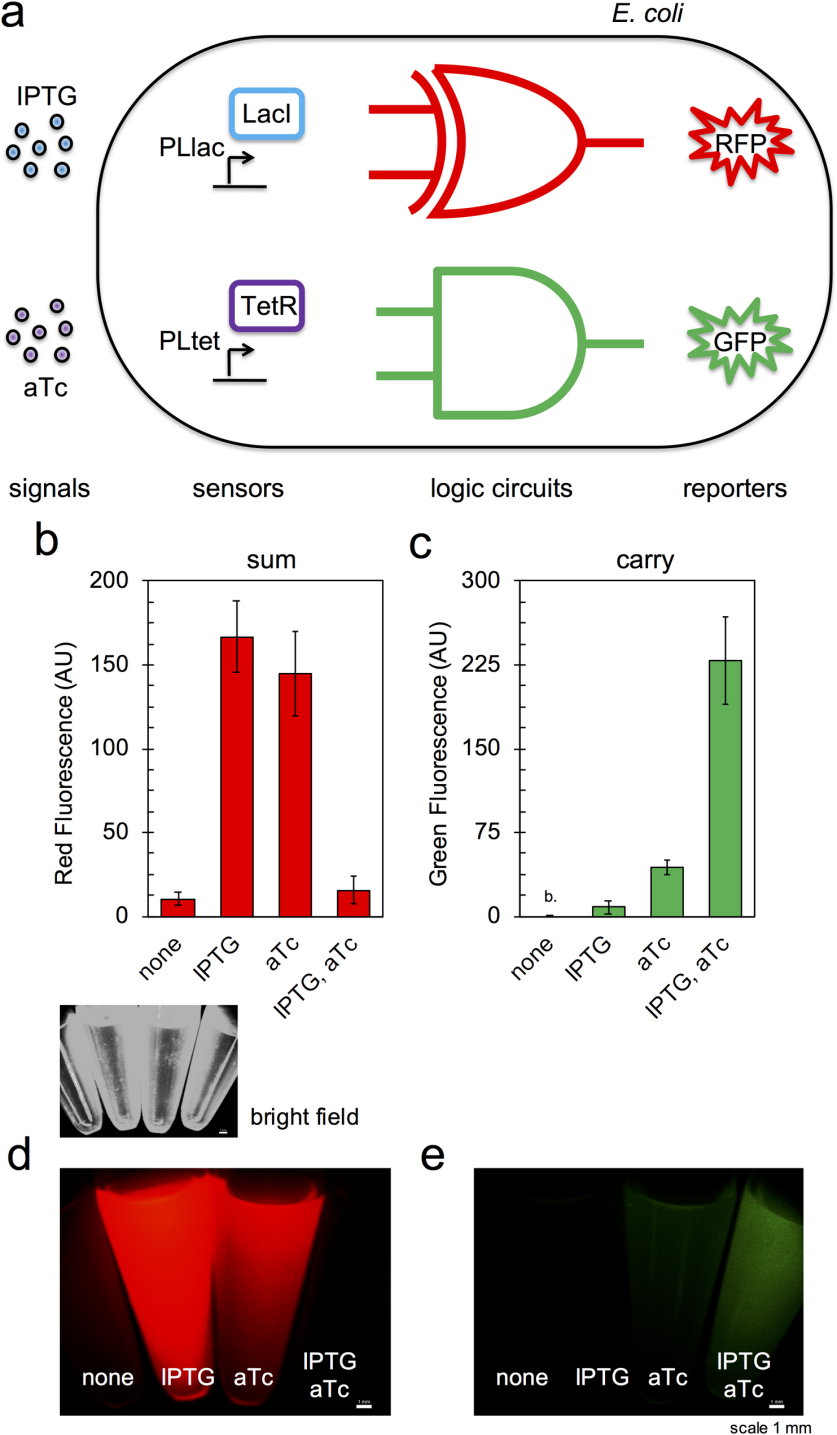
RNA-based half adder. a) Scheme of the computer cell highlighting the different layers. b, c) Normalized red and green fluorescence for each induction condition (IPTG, aTc) when the cell is transformed with the two logic gates. Error bars correspond to standard deviations over three replicates (b. indicates fluorescence below cell autofluorescence). d, e) Images of cell cultures expressing the full system showing red and green fluorescence. Scale, 1 mm. The corresponding bright field image is shown on top.

Finally, we integrated the two modules in a single cell to generate the RNA-based half adder (**Fig 4A**). That is, *E*. *coli* was co-transformed with pRHA12 and pRHA40. Importantly, the riboregulatory systems RAJ11, RAJ12, and RAJ21 were shown computationally, with the NUPACK web application [11], to not suffer cross-talk, *i*.*e*., a given sRNA is not able to release the RBS of a non-cognate 5’ UTR. We measured again red (sum) and green (carry) fluorescence with IPTG and aTc, demonstrating the biological computation (**Fig 4B and 4C**). Nevertheless, we observed that sfGFP was marginally expressed with aTc, perhaps because the transcriptional repression exerted by LacI (less potent than TetR [29]) was slightly abated due to multiple PLlac promoters in the system [40] (section B in **S1 Appendix**). Further work might try to reduce this leakage to enhance the digital behavior of the system. We quantified the performance of the system as the minimal fold change (*f*) between the ON and OFF states. We obtained *f* = 9.4 for mRFP1 (aTc *vs*. IPTG + aTc) and *f* = 5.2 for sfGFP (IPTG + aTc *vs*. aTc). An overall fold change was obtained by averaging geometrically these two values, resulting in *f* = 7.0. Moreover, we inspected the possibility of getting a visual outcome of the circuit computation. For that, we monitored different cell cultures induced with IPTG and aTc with a microscope, showing that the two bits of processed information, corresponding to the sum and the carry, can be easily recognized (**Fig 4D and 4E**).

**Fig 5:**
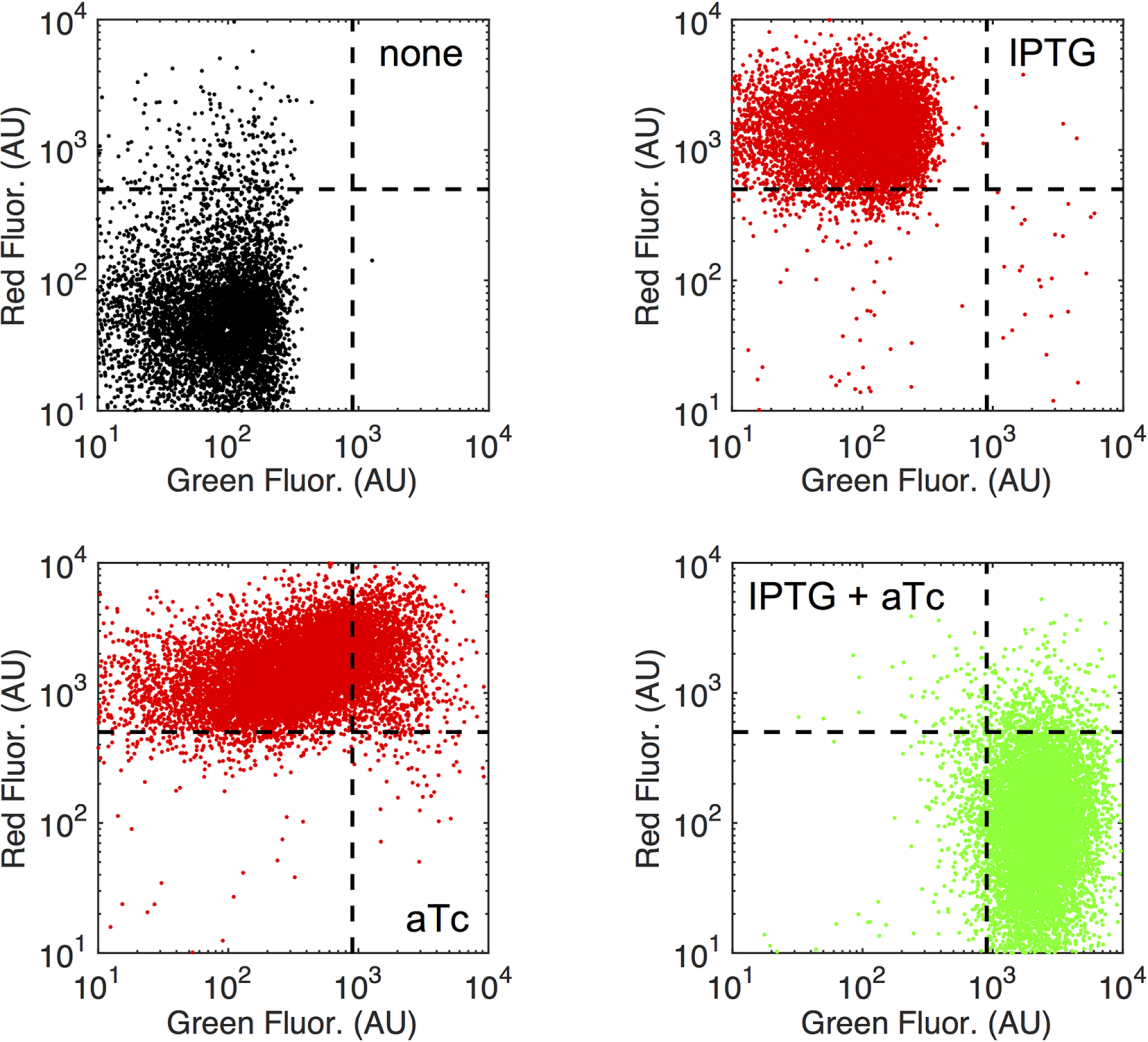
Single cell behavior of the RNA-based half adder. Scatter plots of red and green fluorescence events for each induction condition (IPTG, aTc) when cells harbor the two logic gates. Dashed lines define arbitrary quadrants.

To study whether each *E*. *coli* cell was able to perform the computation (*i*.*e*., respond to the inducers in a relatively homogeneous manner), we further characterized the functionality of our genetic half adder at the single cell level by flow cytometry. Certainly, cell-to-cell variability in gene expression within a clonal population (noise) is an inherent feature of biology [41]. This assay revealed that the whole population significantly shifted its fluorescence in both channels according to the induction condition (Mann-Whitney *U*-tests, *P* ≈ 0; **Fig 5**). Again, we quantified *f* = 16.3 for mRFP1 (now the minimal fold change was in IPTG *vs*. none) and *f* = 4.9 for sfGFP (IPTG + aTc *vs*. aTc) using mean values of fluorescence. The overall fold change was in this case *f* = 8.9. These values are in tune with those reported at the population level. The single cell data also revealed that the slight increase in GFP with only aTc was associated with an increase in cell-to-cell variability regarding sfGFP expression (3.3 times more deviation with aTc than with IPTG). Definitely, more theoretical work is needed to recognize how noise performs in systems of increasing complexity based on intricate transcriptional and post-transcriptional regulation [42].

## Discussion

We have programmed a bacterial cell so that it can perform the binary sum of two bits of information, encoded into the concentrations of IPTG and aTc (signal molecules). The bacterial cell reports the sum of this computation as red fluorescence and the carry as green fluorescence, a sort of minimal biocomputer. To achieve this dynamic behavior, we engineered a genetic system exploiting riboregulation [24]. The whole system consists of four synthetic riboregulators (RAJ11, RAJ11min, RAJ21, and RAJ12), three TFs (LacI, TetR, and cI), and two fluorescent proteins (mRFP1 and sfGFP), which work together within the cell in an articulate manner. Such a system did not require fine-tuning promoters or RBSs to perform as designed, in contrast to what might happen in other cases [43, 44]. Moreover, one important advantage of our designer circuit over the two previous genetic half adders [4, 5] is that the reporter gene conveying the sum is not duplicated. This makes the architecture to be better organized and more scalable, as already pointed out [1].

In addition, the genetic footprint of our designer circuit was greatly reduced thanks to the use of regulatory RNAs, with respect to circuits fully implemented with TFs [6]. The RAJ11, RAJ12, and RAJ21 sRNAs are of 55–71 nucleotides (excluding the terminators), and the RAJ11min sRNA is even of 30 nucleotides. Certainly, the DNA sequence required to encode a protein of average size is much longer. The *cis*-regulating regions at the DNA or RNA levels, by contrast, are of similar size. The PLlac and PLtet promoters are of 54 nucleotides and the 5’ UTRs involved in riboregulation of 52 nucleotides. Beyond this, by only mutating the seed region between the sRNA and the 5’ UTR it is possible to create riboregulatory systems that perform orthogonally *in vivo* [45]. This way, we might easily scale up our designer circuit. Following this strategy, of note, we already created a RAJ11-derived orthogonal system [36].

Definitely, we chose a given molecular implementation, but other implementations might be possible maintaining the same regulatory architecture. As the system does not rely on combinatorial promoters, nothing prevents the use of other input signals (*e*.*g*., endogenous substances of the cell) to perform the computation replacing the PLlac and PLtet promoters by suitable responsive promoters [43, 44]. Alternatively, LacI and TetR might be computationally redesigned to sense new compounds [46]. The riboregulatory mode, here characterizing an internal layer of gene expression activation, is also flexible. *Cis*-repression of translation might occur by trapping the start codon, instead of the RBS, in the 5’ UTR structure [25]. More distinctly, the activation might be transcriptional with sRNAs that act in *trans* as anti-terminators [14]. In addition to LacI and TetR (working in the sensory layer), our system also involves the TF cI to implement an internal repression in the XOR logic gate. We tried to implement this repression by antisense RNA [47] or CRISPR interference [15], without successful results (section C in **S1 Appendix**); arguably, because the expression of mRFP1 was from a high-copy plasmid. This reveals the necessity of pursuing the development of novel RNA-based mechanisms and circuits. All in all, our genetic implementation of an ALU promises to be important in the future to develop smart cells (*e*.*g*., diagnostic bacteria for clinical use) that can make appropriate decisions after certain processing (computation) of the signals perceived from the medium [48].

## Materials and methods

### Regulatory sequences

Synthetic PL-based promoters regulated by the TFs LacI and TetR [29] were used as elements to sense the input signals (IPTG and aTc). Riboregulatory sequences (sRNAs and 5’ UTRs) of systems RAJ11, RAJ12, and RAJ21 were obtained from previous work [31], as well as the sequences of system RR12 [24]. A minimal version of the sRNA RAJ11 was designed by removing the nucleotides not contributing to the intermolecular interaction. The structural models of these systems are shown in **S2 Appendix**. The PR promoter and a codon-optimized version of the TF cI from λ phage [37] were also used.

### Plasmid construction

Six plasmids were characterized in this work: pRAJ11, pRHA12, pRHA25, pRHA36, pRHA37, and pRHA40. First, pRAJ11 (ampR, pUC ori) and pRAJ12 (kanR, pSC101m ori) were taken from previous work [31]. pRHA12 was constructed by removing the mRFP1 gene from pRAJ12. pRAJ11 expresses in a controlled way GFPmut3b and pRHA12 sfGFP. Moreover, pRHA25 (ampR, pUC ori) was synthesized by IDT. This expresses in a controlled way mRFP1. pRHA36 was constructed by inserting in pRHA25 an expression cassette of cI regulated by ribosystem RR12 (synthesized by IDT), also changing the J23119 promoter by the PR promoter. pRHA37 was constructed by removing the expression cassette of cI from pRHA36. Finally, pRHA40 was constructed by inserting in pRHA37 an expression cassette of cI regulated by ribosystem RAJ21 (synthesized by IDT). See sequences in **S3 Appendix**.

### Strains, cell cultures, and reagents

For cloning purposes, *E*. *coli* Dh5α was used following standard procedures [49]. To express the circuits, *E*. *coli* MG1655-Z1 (F^-^, λ^-^, *rph-1*, *lacI*^q^, PN25:*tetR*, Sp^R^) was used (*i*.*e*., a strain that is *lacI*^+^ and *tetR*^+^). LB medium was used for overnight cultures, while M9 minimal medium (1x M9 salts, 2 mM MgSO_4_, 0.1 mM CaCl_2_, 0.4% glucose, 0.05% casamino acids, and 0.05% thiamine) for characterization cultures. IPTG was used at the concentration of 1 mM and aTc at 100 ng/mL. Ampicillin and kanamycin were used as antibiotics at the concentration of 50 μg/mL. Compounds provided by Sigma-Aldrich.

### Fluorescence quantification

Cultures (2 mL) inoculated from single colonies (three replicates) were grown overnight in LB medium at 37°C and 200 rpm. Cultures were then diluted 1:200 (1:100 in the case of cells expressing pRHA40) in M9 minimal medium (2 mL) with appropriate inducers (IPTG, aTc) and were grown for 5–8 h, depending on the genetic system and induction condition, at 37°C and 200 rpm to reach an OD_600_ around 0.5. Cultures were then used to load the wells (200 μL) of the microplate (96 wells, black, clear bottom; Corning). This was assayed in a fluorometer (Perkin Elmer Victor X5) to measure absorbance (600 nm absorbance filter), green fluorescence (485/14 nm excitation filter, 535/25 nm emission filter), and red fluorescence (570/8 nm excitation filter, 610/10 nm emission filter). Mean background values of absorbance and fluorescence, corresponding to M9 minimal medium, were subtracted to correct the readouts. Normalized fluorescence was calculated as the ratio of fluorescence and absorbance. The mean value of normalized fluorescence corresponding to cells transformed with control plasmids was then subtracted to obtain a final estimate of expression.

### Culture imaging

A culture (2 mL) inoculated from a single colony was grown overnight in LB medium at 37°C and 200 rpm. The culture was then diluted 1:100 in M9 minimal medium (2 mL) and was grown for 5 h at 37°C and 220 rpm to reach exponential phase. The culture was then diluted 1:40 in M9 minimal medium (2 mL) with appropriate inducers (IPTG, aTc) and was grown for 8 h at 37°C and 220 rpm to reach an OD_600_ around 0.7. 200 μL of each culture were transferred to small tubes. Culture images were acquired with a light microscope (Leica DFC7000T) with the fluorescence filters for GFP and DsRed. Exposition parameters were manually adjusted to enhance the quality of the image.

### Flow cytometry

A culture (2 mL) inoculated from a single colony was grown overnight in LB medium at 37°C and 200 rpm. The culture was then diluted 1:100 in M9 minimal medium (2 mL) and was grown for 5 h at 37°C and 200 rpm to reach exponential phase. The culture was then diluted 1:50 in M9 minimal medium (200 μL) and placed in a microplate with appropriate inducers (IPTG, aTc) and was grown for 5 h at 37°C and 1,000 rpm in a plate shaker (Biosan PST-60HL). Cultures were spun down at 13,000 rpm for 2 min and resuspended in PBS (2 mL). Fluorescence was measured with a flow cytometer (BD LSRFortessa, lasers of 488 nm and 561 nm) with the emission filters for GFP (530/30 nm) and DsRed (585/15 nm). Events were then gated and compensated (~15,000 after this process). The mean value of the autofluorescence of the cells was subtracted in each channel to obtain a final estimate of expression.

## Supporting information

S1 Appendix Additional results. It contains further fluorescence data of the engineered circuits.
(PDF)

S2 Appendix Structures. It contains the intra- and intermolecular secondary structures of the riboregulatory systems.
(PDF)

S3 Appendix Sequences. It contains the precise nucleotide sequences of the regulatory elements and engineered circuits.
(PDF)
