# Supplementary material for: Binary addition in a living cell based on riboregulation
Source: PLoS Genet. 2018 Jul 19;14(7):e1007548. doi: 10.1371/journal.pgen.1007548 (PMC6067762; doi:10.1371/journal.pgen.1007548)
Supplement: S1 Appendix — It contains further fluorescence data of the engineered circuits. (PDF) [file pgen.1007548.s001.pdf]

S1 Appendix

A) Apparent fluorescence of the genetic systems

In the main manuscript, we show the fluorescence values after normalization by absorbance and subsequent subtraction of the autofluorescence of the cells. See also in the following picture the normalized green fluorescence for each induction condition (IPTG, aTc) when the circuit shown in Fig. 1a was implemented with the RAJ11 sRNA and GFPmut3b.

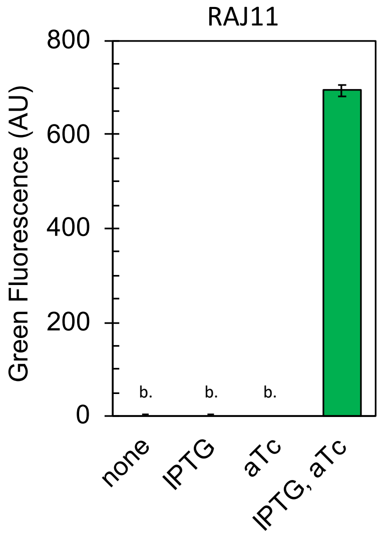

In the case of GFP, we observed that the autofluorescence of the cells was comparable to the apparent fluorescence derived from the uninduced genetic systems, in agreement with previous results [1, 2]. In other words, GFP was tightly repressed due to the RNA structure in the 5' UTR and cells by themselves had a marginal green fluorescence. In the following pictures (data corresponding to AND logic gates implemented with riboregulators RAJ11 or RAJ12), the horizontal line denotes the average value of green fluorescence, normalized by absorbance, of cells carrying plasmids that do not express GFP.

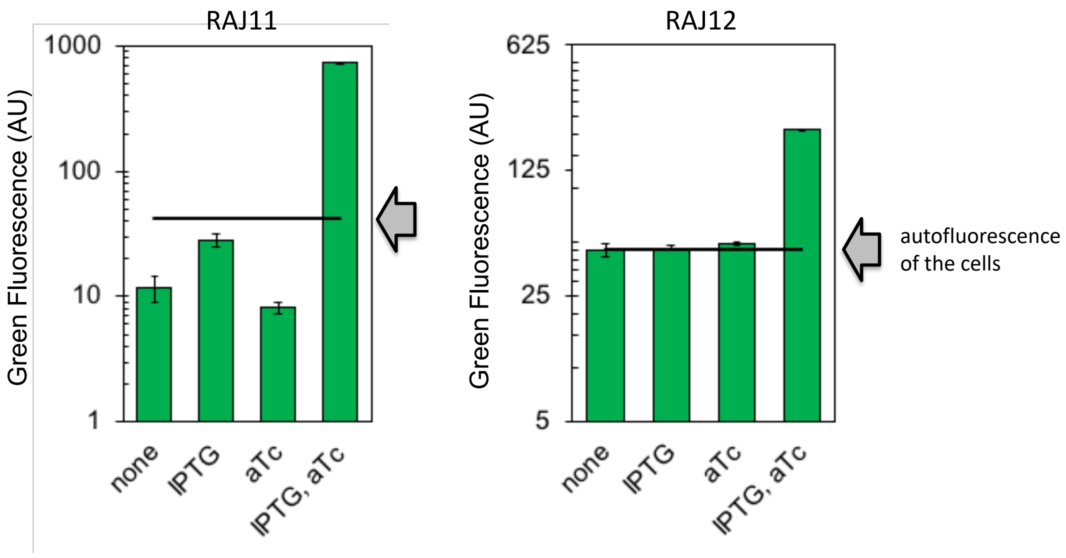

However, in the case of RFP, we observed that the autofluorescence of the cells was irrelevant. The reported values of apparent fluorescence for cells that do not express RFP were even lower than the values for the culture medium. This way, in the following pictures (data corresponding to XOR logic gates implemented with riboregulators RR12 or RAJ21), the horizontal line, denoting the average value of red fluorescence of cells that do not express RFP, is in 0.

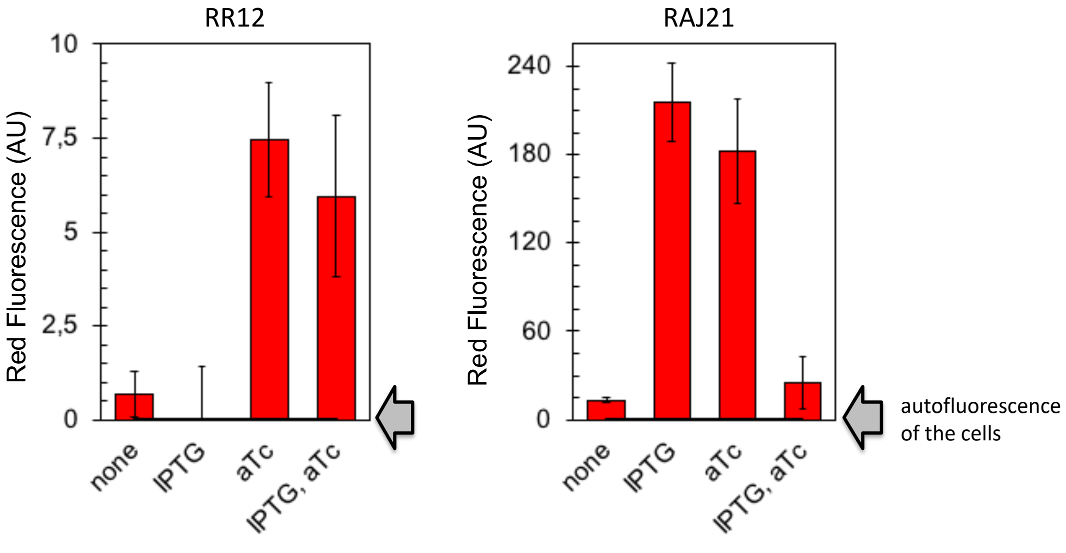

The fluorescence values of the system implemented with riboregulator RR12 (< 10) are much lower than the fluorescence values of a constitutive expression system, indicating that RFP is marginally expressed in this case.

B) Analysis of expression leakages in the circuits

The riboregulatory system RAJ12 was functional (AND logic behavior) when it was expressed from a medium copy number plasmid (pSC101m ori). However, when pRHA12 (pSC101m ori) was co-transformed with pRHA40 (pUC ori) there was a marginal GFP expression with only aTc, as shown in the main manuscript. This revealed that there was GFP production even without IPTG, *i.e.*, when there was RAJ12 sRNA in abundance and apparently no mRNA. This hence suggested that the PLlac promoter, despite the repression exerted by LacI (constitutively expressed in the strain of use), was marginally active in this scenario of co-transformation (at least, this occurs with the 5' UTR sequence of system RAJ12). Arguably, the presence of additional PLlac promoters in pRHA40 might affect the expression coming from pRHA12 by titrating LacI proteins, something that might also occur with the PLtet promoter [3]. This might be reverted by increasing the expression of LacI (note that with *lacI*^{q} we have ~100 molecules/cell with respect to ~10 molecules/cell in the wild-type scenario [4], which still may be low for some applications). Supporting this hypothesis is the fact that the dynamic range of the PLtet promoter decreases with plasmid copy number [5] (a feature that might also be applicable to the PLlac promoter).

To get further insight (and discard eventual non-cognate sRNA-mRNA interactions), we studied the GFP and RFP expressions from cells co-transformed with pRHA12 and pRHA36. As expected, GFP was also marginally expressed with only aTc, as shown in the following picture. Experimentally, we already proved that the RAJ11 sRNA was not able to directly activate the RAJ12 5' UTR [1]. And our new results indicate that the marginal GFP expression with aTc cannot be attributed to the effect of the RAJ21 or RR12 sRNAs. The investigation of this issue certainly goes

beyond the scope of this paper, so further work is required (perhaps by performing a robustness test in which the half-adder performance is systematically checked in the absence of one-by-one key components) to fully recognize the underlying mechanistic basis and then act on it to enhance the digital behavior of our engineered AND gate.

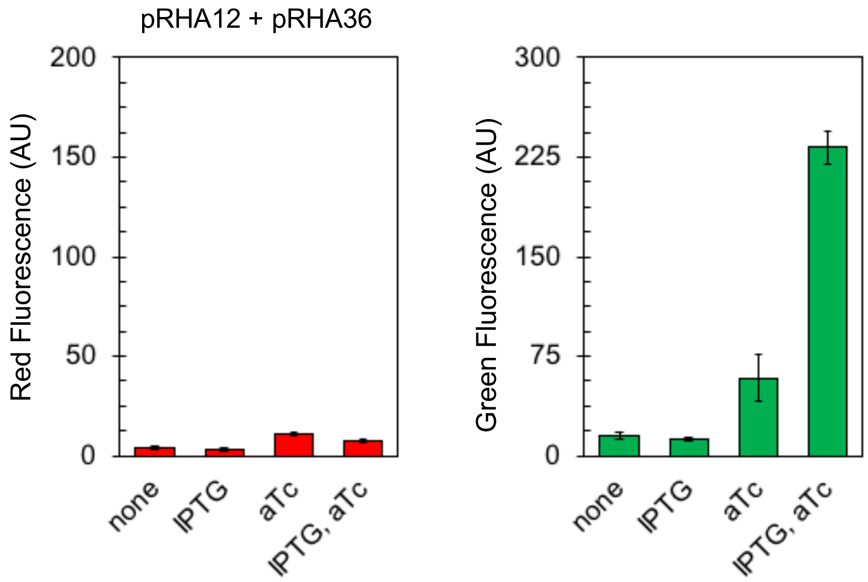

Furthermore, in cells transformed with pRHA36, we observed that RFP was expressed at similar levels for the aTc only and aTc + IPTG conditions. This might reflect a marginal expression of the RFP-coding mRNA, which would be translated into protein by the action of the RAJ11 sRNA, which is expressed with aTc.

C) Different implementations tried to get a XOR logic gate

We first tried to repress the RFP expression with an antisense RNA (asRNA) once the 5’ UTR is structurally rearranged by the action of the RAJ11 or RAJ11min sRNAs [6]. For that, we introduced a linker between the first (start) and second codons of the protein, whose sequence was `TCTAGATTGTTATCCGCTCACAAT`. Then, the designed asRNA had to hybridize in this region. The sequence assayed was `GAATTGTG AGCGGATAACAATTTCACACATCTAGGCATTTTCTGTTGGGCCATTGCATTGCCACT GATTTTCCAACATATAAAAAGACAAGCCCGAACAGTCGTCCGGGCTTTTTTT`, where the MicC scaffold is shown in gray. The asRNA was expressed from the same plasmid that expresses RFP with the PLtet/lac promoter (Registry of Standard Biological Parts #K091101), a version of the PLtet promoter that also includes a lac operator. However, this system was not able to apparently repress RFP, at least when the target is expressed from a high copy number plasmid. Also, in this system, the RAJ11 sRNA was expressed with the PN25tet promoter (Expressys), which was revealed weaker than the PLtet promoter.

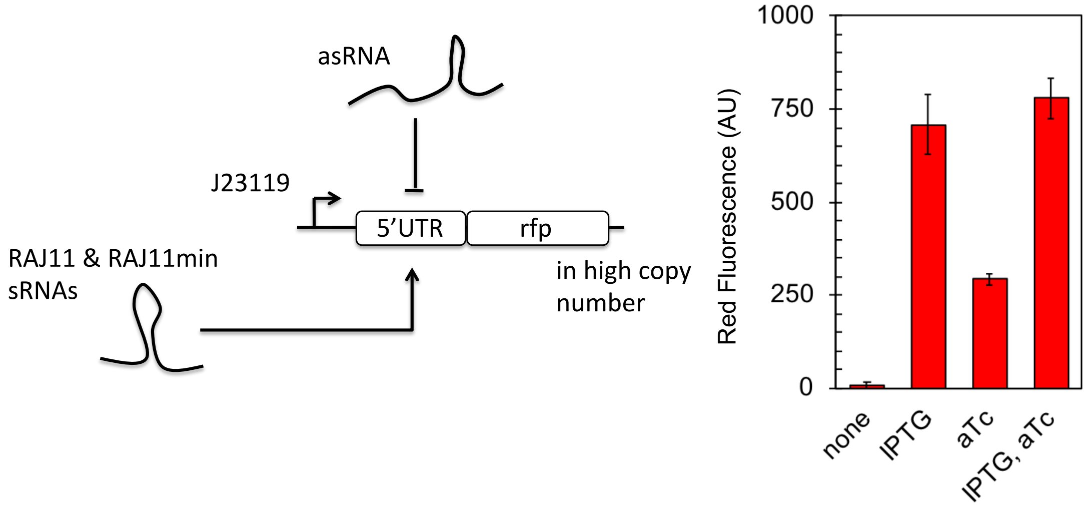

Then, we decided to use CRISPR interference [7]. For that, we expressed a guide RNA with the PLlac promoter from the same plasmid that expresses RFP,

together with dCas9 with the PLtet promoter from the pdCas9-bacteria plasmid (Addgene #44249). The sequence of the guide RNA, acting on the RFP coding region, was AACTTTCAGTTTAGCGGTCTGTTTTAGAGCTAGAAATAGCAAGTTAAAATAAGGCTAGTCCG, where the dCas9 recognition hairpin is shown in gray. This repression system only reduced in 2.1 fold the RFP expression induced by the action of the RAJ11 and RAJ11min sRNAs in a high copy number plasmid.

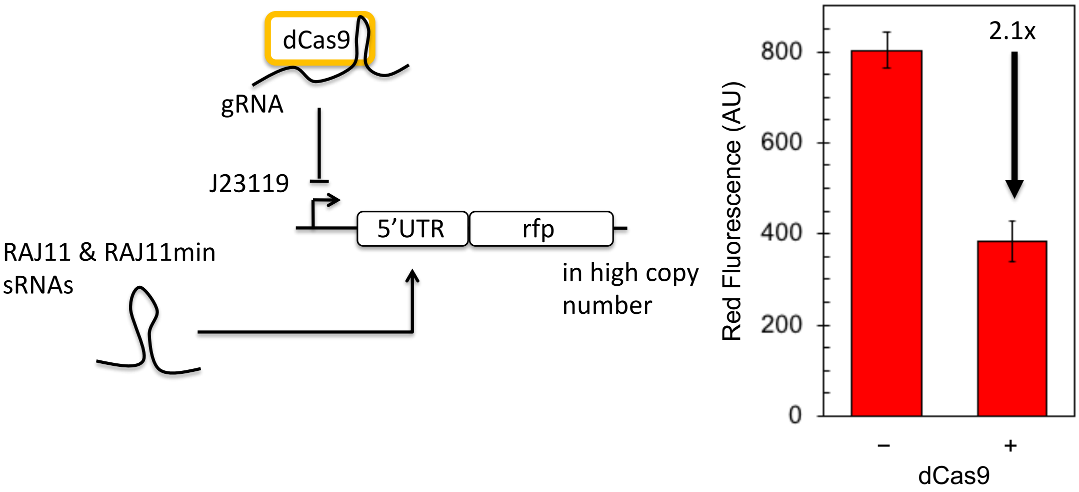

Finally, we replaced the J23119 promoter by the PR promoter to use the transcriptional repressor cI, which is very strong [8]. This repressor was indeed able to inhibit substantially (7.7 fold change) the RFP expression induced by the action of the sRNAs in a high copy number plasmid.

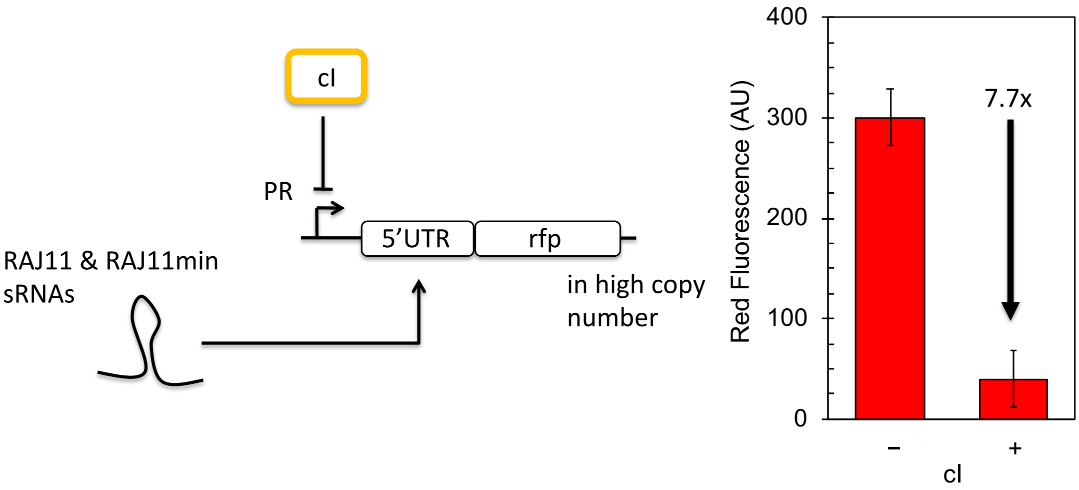

References

[1] Rodrigo G, Landrain TE, Jaramillo A (2012) De novo automated design of small RNA circuits for engineering synthetic riboregulation in living cells. *Proc Natl Acad Sci USA* 109: 15271-15276.

[2] Green AA, Silver PA, Collins JJ, Yin P (2014) Toehold switches: de-novo-designed regulators of gene expression. *Cell* 159: 925-939.

[3] Lee TH, Maheshri N (2012) A regulatory role for repeated decoy transcription factor binding sites in target gene expression. *Mol Syst Biol* 8: 576.

[4] Glascock CB, Weickert MJ (1998) Using chromosomal lacIQ1 to control expression of genes on high-copy-number plasmids in Escherichia coli. *Gene* 223: 221-231.

[5] Lutz R, Bujard H (1997) Independent and tight regulation of transcriptional units in Escherichia coli via the LacR/O, the TetR/O and AraC/I1-I2 regulatory elements. *Nucleic Acids Res* 25: 1203-1210.

[6] Na D, Yoo SM, Chung H, Park H, Park JH, Lee SY (2013) Metabolic engineering of Escherichia coli using synthetic small regulatory RNAs. *Nat Biotechnol* 31: 170-174.

[7] Qi LS, Larson MH, Gilbert LA, Doudna JA, Weissman JS, Arkin AP, Lim WA (2013) Repurposing CRISPR as an RNA-guided platform for sequence-specific control of gene expression. *Cell* 152: 1173-1183.

[8] Rosenfeld N, Young JW, Alon U, Swain PS, Elowitz MB (2005) Gene regulation at the single-cell level. *Science* 307: 1962-1965.
