# Supplementary figures and images for: Binary addition in a living cell based on riboregulation
Source: PLoS Genet. 2018 Jul 19;14(7):e1007548. doi: 10.1371/journal.pgen.1007548 (PMC6067762; doi:10.1371/journal.pgen.1007548)

## S2 Appendix

### System RAJ11

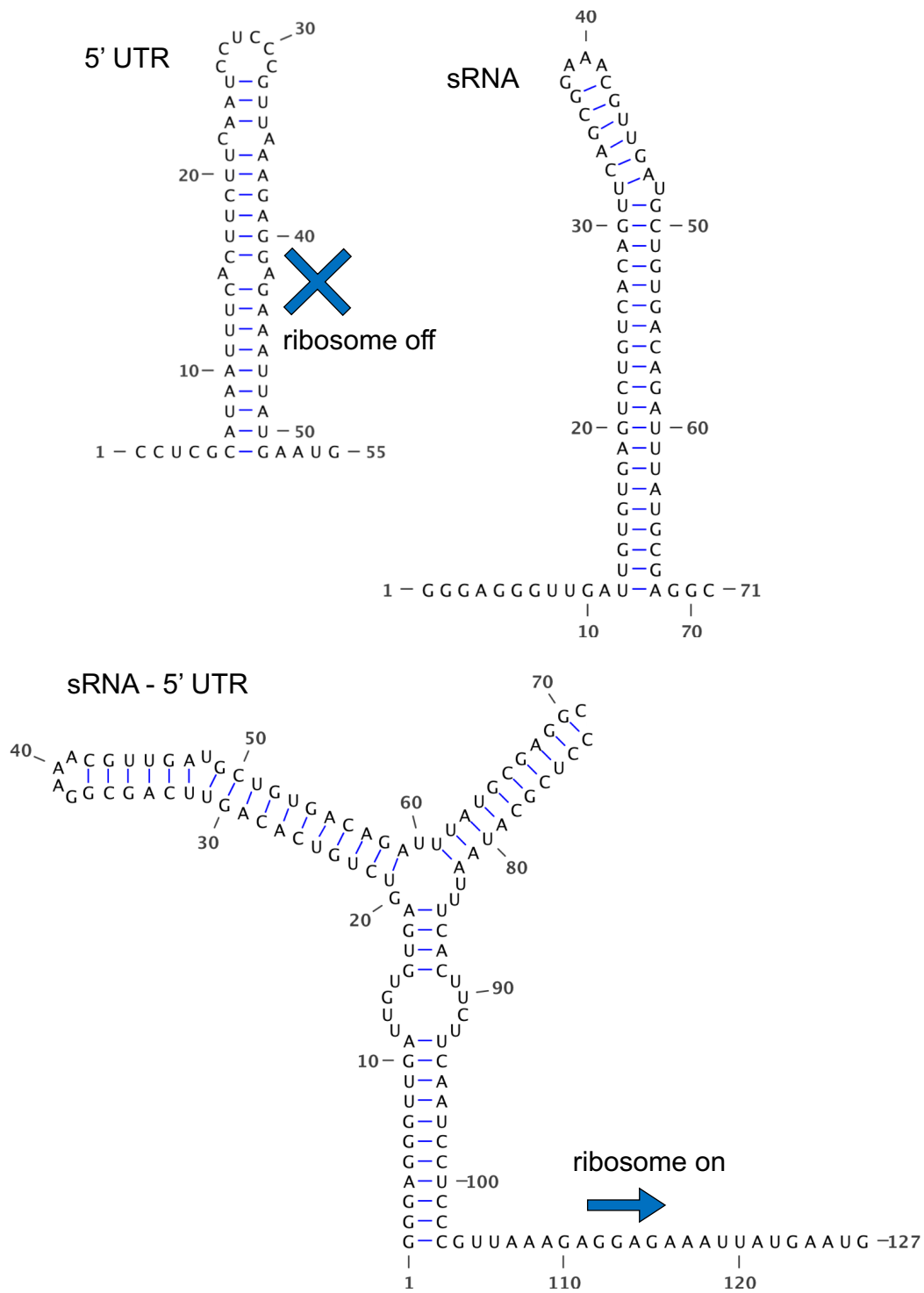

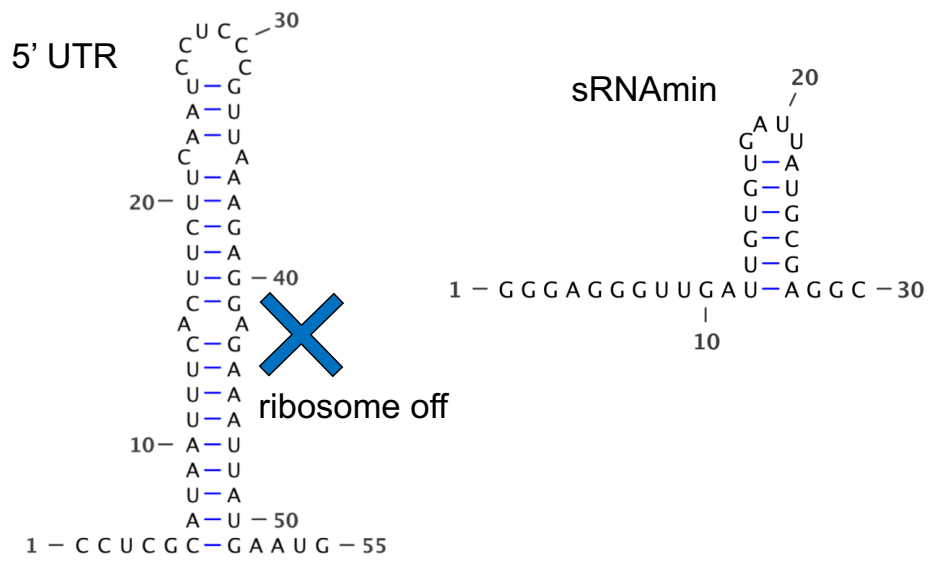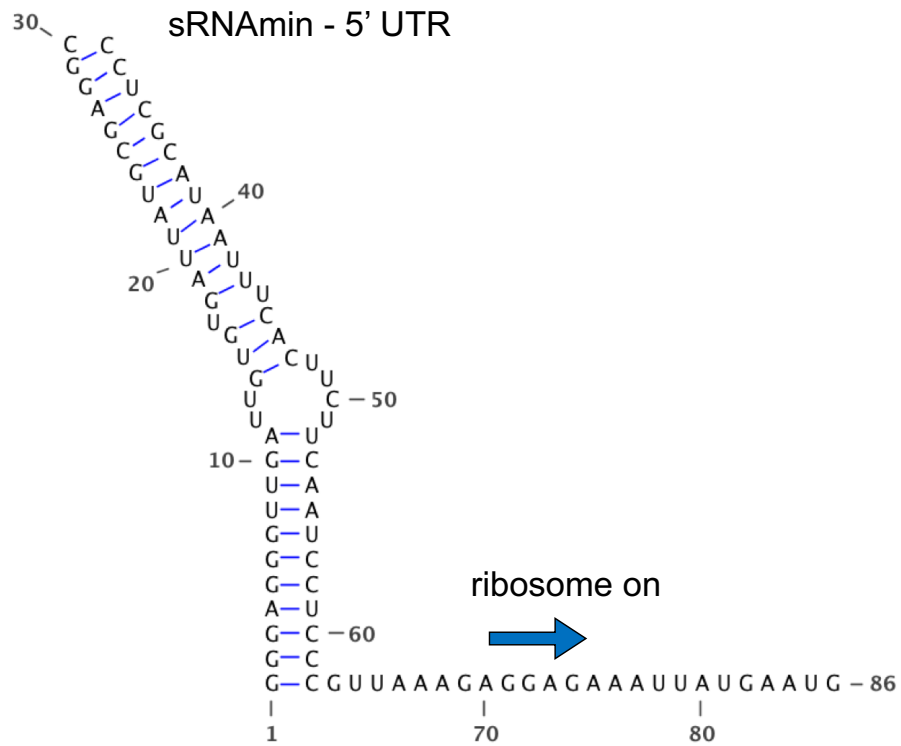



## System RAJ12

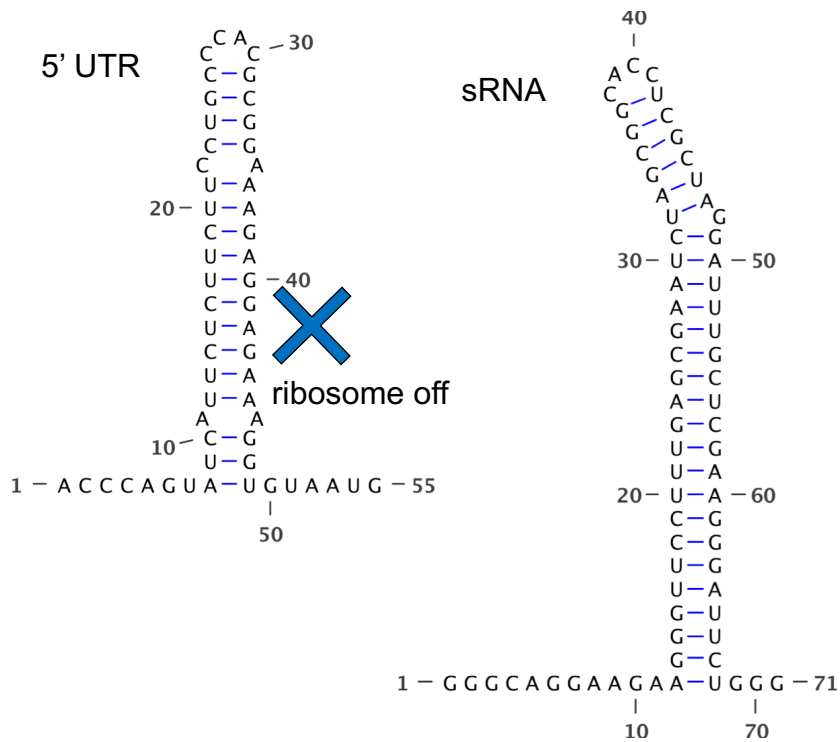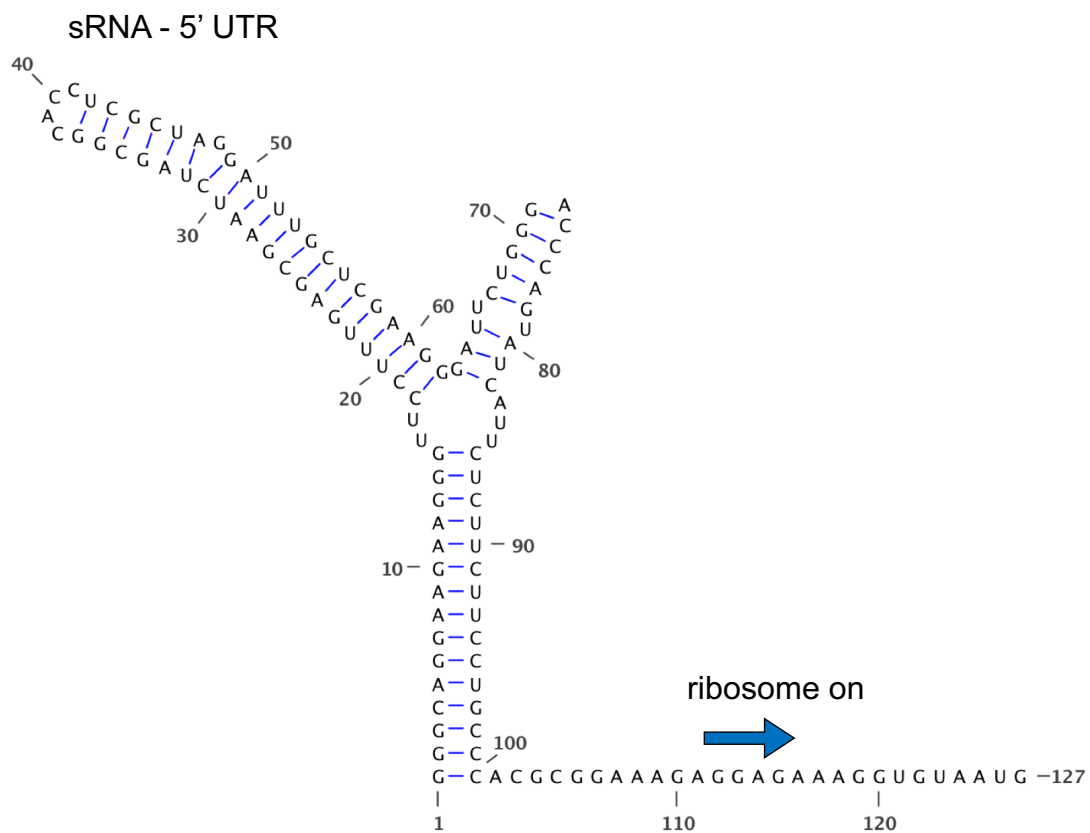

## System RAJ21

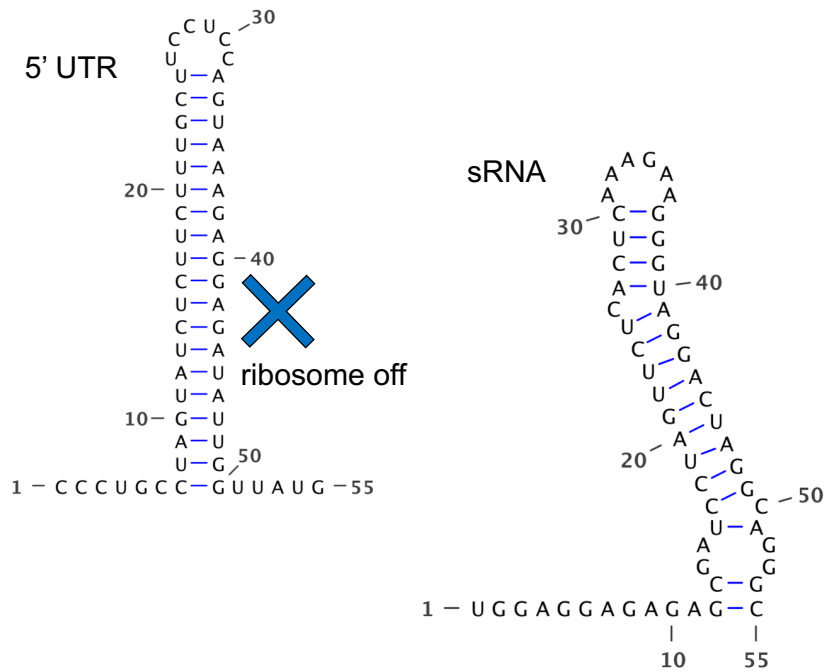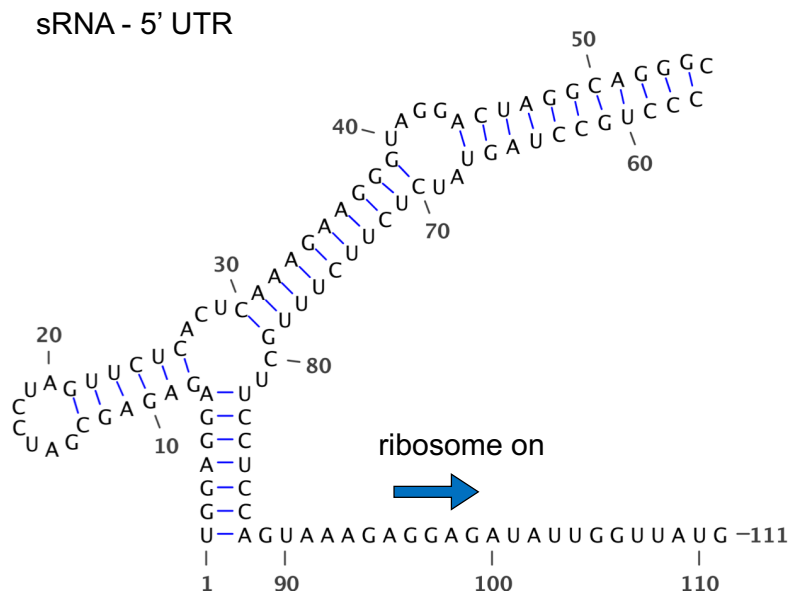

## System RR12

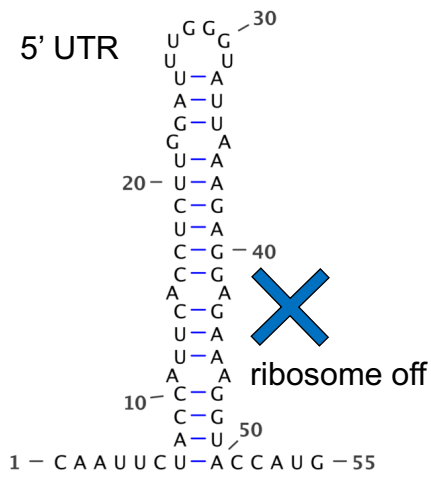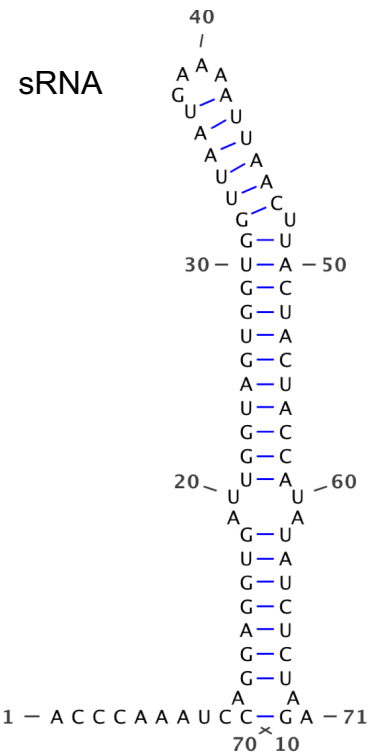

### sRNA - 5' UTR

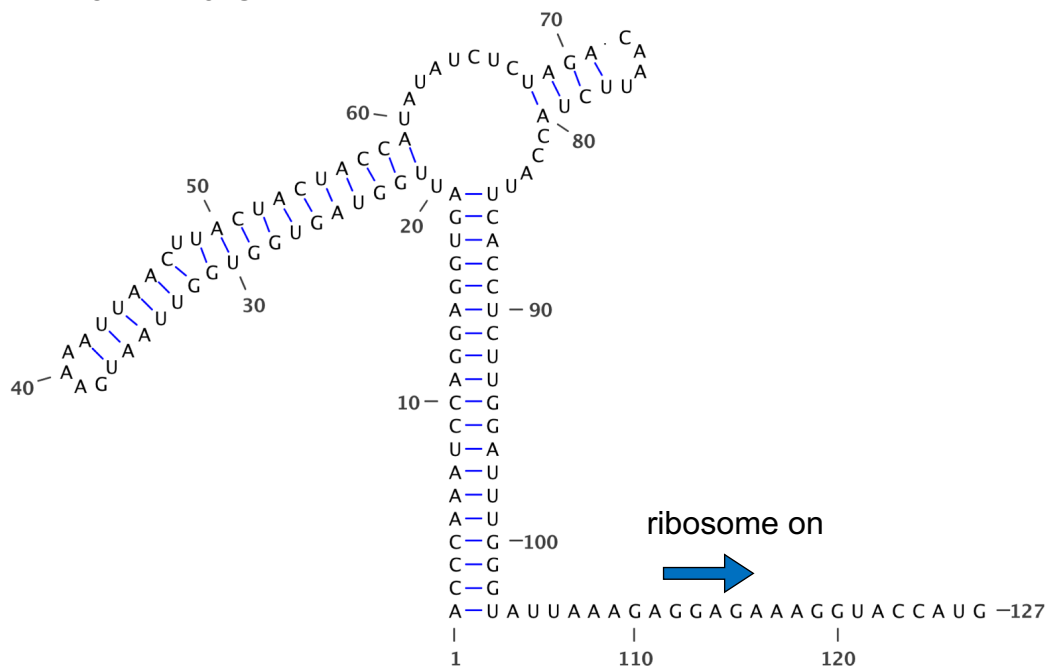

Supplement: S2 Appendix — It contains the intra- and intermolecular secondary structures of the riboregulatory systems. (PDF) [file pgen.1007548.s002.pdf]
