# Supplementary material for: Binary addition in a living cell based on riboregulation
Source: PLoS Genet. 2018 Jul 19;14(7):e1007548. doi: 10.1371/journal.pgen.1007548 (PMC6067762; doi:10.1371/journal.pgen.1007548)
Supplement: S3 Appendix — It contains the precise nucleotide sequences of the regulatory elements and engineered circuits. (PDF) [file pgen.1007548.s003.pdf]

S3 Appendix

In this file, we present the sequences of all genetic elements used to engineer the RNA-based half-adder, as well as the sequences of the final constructs.

Regulatory RNAs

>5’UTR_RAJ11 (with start codon)

CCTCGCATAATTTCACTTCTTCAATCCTCCCGTTAAAGAGGAGAAATTATGAATG

>RAJ11

GGGAGGGTTGATTGTGTGAGTCTGTCACAGTTCAGCGGAAACGTTGATGCTGTGACAGATTTATGCGAGGC

>RAJ11min

GGGAGGGTTGATTGTGTGATTATGCGAGGC

>5’UTR_RAJ12 (with start codon)

ACCCAGTATCATTCTCTTCTTCCTGCCCACGCGGAAAGAGGAGAAAGGTGTAATG

>RAJ12

GGGCAGGAAGAAGGGTTCCTTTGAGCGAATCTAGCGGCACCTCGCTAGGATTTGCTCGAAGGGATTCTGGG

>5’UTR_RAJ21 (with start codon)

CCCTGCCTAGTATCTCTTCTTTGCTTCCTCCAGTAAAGAGGAGATATTGGTTATG

>RAJ21

TGGAGGAGAGAGCGATCCTAGTTCTCACTCAAAGAAGGGTAGGACTAGGCAGGGC

>5’UTR_RR12 (with start codon)

CAATTCTACCATTCACCTCTTGGATTTGGGTATTAAAGAGGAGAAAGGTACCATG

>RR12

ACCCAAATCCAGGAGGTGATTGGTAGTGGTGGTTAATGAAAATTAACTTACTACTACCATATATCTCTAGA

Promoters

>PLlacO1

AATTGTGAGCGGATAACAATTGACATTGTGAGCGGATAACAAGATACTGAGCAC

>PLlacO12

ATAAATGTGAGCGGATAACATTGACATTGTGAGCGGATAACAAGATACTGAGCAC

>PLtetO1

TCCCTATCAGTGATAGAGATTGACATCCCTATCAGTGATAGAGATACTGAGCAC

>PLtetO12

ACTCTATCATTGATAGAGTTTGACATCCCTATCAGTGATAGAGATACTGAGCAC

>J23119

TTGACAGCTAGCTCAGTCCTAGGTATAATGCTAGC

>PR(lambda)

TAACACCGTGCGTGTTGACTATTTTACCTCTGGCGGTGATAATGGTTGC

Terminators

>TtonB

CACCGAAATTCAGTAAGCAGAAAGTCAAAAGCCTCCGACCGGAGGCTTTTGACTATTACTCAACAGGTAAGGCGC
GAGGTTTTC

>TtonB-deriv

ATTAGCAGAAAGTCAAAAGCCTCCGACCGGAGGCTTTTGACTAAAACTTCCCTTGGGGTTATCATTGGG

>T500

CAAAGCCCGCCGAAAGGCGGGCTTTTCTGT

>TrrnC

ATCCTTAGCGAAAGCTAAGGATTTTTTTT

>TM13central-min

GGCTCCTTTTGGAGCCTTTTTTTTT

>B1002

CGCAAAAAACCCCGCTTCGGCGGGGTTTTTTCGC

Proteins

>sfGFP (without start and stop codons)

cgtaaaggcgaagagctgttcactggtgtcgtccctattctggtggaactggatggtgatgtcaacggtcataag
ttttccgtgcgtggcgagggtgaaggtgacgcaactaatggtaaactgacgctgaagttcatctgtactactggt
aaactgccggtaccttggccgactctggtaacgacgctgacttatggtgttcagtgctttgctcgttatccggac
catatgaagcagcatgacttcttcaagtccgccatgccggaaggctatgtgcaggaacgcacgatttcctttaag
gatgacggcacgtacaaaacgcgtgcggaagtgaaatttgaaggcgataccctggtaaaccgcattgagctgaaa
ggcattgactttaaagaagacggcaatatcctgggccataagctggaatacaattttaacagccacaatgtttac
atcaccgccgataaacaaaaaaatggcattaaagcgaattttaaaattcgccacaacgtggaggatggcagcgtg
cagctggctgatcactaccagcaaaacactccaatcggtgatggtcctgttctgctgccagacaatcactatctg
agcacgcaaagcgttctgtctaaagatccgaacgagaaacgcgatcatatggttctgctggagttcgtaaccgca
gcgggcatcacgcatggtatggatgaactgtacaaa

>mRFP1 (without start and stop codons)

gcttcctccgaagacgttatcaaagagttcatgcgtttcaaagttcgtatggaaggttccgttaacggtcacgag
ttcgaaatcgaaggtgaaggtgaaggtcgtccgtacgaaggtacccagaccgctaaactgaaagttaccaaaggt
ggtccgctgccgttcgcttgggacatcctgtccccgcagttccagtacggttccaaagcttacgttaaacacccg
gctgacatcccggactacctgaaactgtccttcccggaaggtttcaaatgggaacgtgttatgaacttcgaagac
ggtggtgttgttaccgttacccaggactcctccctgcaagacggtgagttcatctacaaagttaaactgcgtggt
accaacttcccgtccgacggtccggttatgcagaaaaaaaccatgggttgggaagcttccaccgaacgtatgtac
ccggaagacggtgctctgaaaggtgaaatcaaaatgcgtctgaaactgaaagacggtggtcactacgacgctgaa
gttaaaaccacctacatggctaaaaaaccggttcagctgccgggtgcttacaaaaccgacatcaaactggacatc
acctcccacaacgaagactacaccatcgttgaacagtacgaacgtgctgaaggtcgtcactccaccggtgct

>cI(lambda) (without start and stop codons)

TCAACCAAGAAAAAGCCACTTACGCAGGAGCAACTGGAAGATGCTCGTCGTTTGAAAGCCATCTACGAGAAAAAG
AAAAATGAACTTGGCTTATCCCAAGAGTCTGTAGCGGATAAAATGGGTATGGGGCAGAGTGGAGTGGCGCGCTT
TTTAACGGTATTAATGCTTTAAACGCATACAATGCAGCATTATTAGCAAAAATCCTGAAAGTAAGTGTTGAAGAG
TTCAGTCCTTCCATTGCTCGTGAAATCTACGAGATGTATGAGGCGGTTTCTATGCAACCGTCCTTACGTAGCGAG
TATGAATACCCGGTGTTCTCCCACGTTCAAGCGGGCATGTTTAGTCCCGAACTTCGTACGTTTACAAAGGGCGAC
GCGGAGCGTTGGGTGAGTACCACGAAAAAGGCATCTGACTCTGCTTTTTGGCTGGAGGTAGAAGGTAACTCCATG
ACGGCACCCACTGGAAGTAAACCCTCATTCCCGGATGGGATGCTGATTTTGGTTGACCCCGAACAGGCTGTAGAA
CCAGGGGATTTTTGCATCGCCCGTCTTGGAGGCGATGAGTTTACCTTTAAGAAACTTATCCGTGACAGTGGACAA
GTTTTTTTGCAACCGCTTAATCCGCAGTATCCCATGATTCCGTGCAATGAATCCTGTAGCGTGGTGGGGAAAGTA
ATTGCGAGCCAGTGGCCCGAAGAAACATTCGGT

AND gate (with GFP)

rev(B1002) + NheI + rev(RAJ12) + rev(PLtetO1) + AvrII + NsiI + PLlacO12 +

5’UTR_RAJ12 + sfGFP + SpeI + PstI TtonB-deriv

GCGAAAAAACCCCGCCGAAGCGGGGTTTTTTGCGgctagcCCCAGAATCCCTTCGAGCAAATCCTAGCGAGGTGC
CGCTAGATTCGCTCAAAGGAACCCTTCTTCCTGCCCGTGCTCAGTATCTCTATCACTGATAGGGATGTCAATCTC
TATCACTGATAGGGAcctaggatgcatATAAATGTGAGCGGATAACATTGACATTGTGAGCGGATAACAAGATAC
TGAGCACACCCAGTATCATTCTCTTCTTCCTGCCCACGCGGAAAGAGGAGAAAGGTGTAATGACTAGCcgtaaag
gcgaagagctgttcactggtgtcgtccctattctggtggaactggatggtgatgtcaacggtcataagttttccg
tgcgtggcgagggtgaaggtgacgcaactaatggtaaactgacgctgaagttcatctgtactactggtaaactgc
cggtaccttggccgactctggtaacgacgctgacttatggtgttcagtgctttgctcgttatccggaccatatga
agcagcatgacttcttcaagtccgccatgccggaaggctatgtgcaggaacgcacgatttcctttaaggatgacg
gcacgtacaaaacgcgtgcggaagtgaaatttgaaggcgataccctggtaaaccgcattgagctgaaaggcattg
actttaaagaagacggcaatatcctgggccataagctggaatacaattttaacagccacaatgtttacatcaccg
ccgataaacaaaaaaatggcattaaagcgaattttaaaattcgccacaacgtggaggatggcagcgtgcagctgg
ctgatcactaccagcaaaacactccaatcggtgatggtcctgttctgctgccagacaatcactatctgagcacgc
aaagcgttctgtctaaagatccgaacgagaaacgcgatcatatggttctgctggagttcgtaaccgcagcgggca
tcacgcatggtatggatgaactgtacaaaTGATGATactagtAGCGGCCGctgcagGAGTCACTAAGGGTTAGTT
AGTTAGATTAGCAGAAAGTCAAAAGCCTCCGACCGGAGGCTTTTGACTAAAACTTCCCTTGGGGTTATCATTGGG

XOR gate (with RFP)

EcoRI + rev(TtonB) + rev(mRFP1) + SpeI + rev(5’UTR_RAJ11) + rev(PR(lambda)) + PLlacO1 + RAJ11min + TrrnC + XbaI + PLtetO1 + RAJ11 + TM13central-min + BglII + PstI + rev(B1002) + rev(RAJ21) + rev(PLtetO12) + XhoI + PLlacO12 + 5’UTR_RAJ21 + ApaI + cI(lambda) + T500 + BamHI

gaattcGAAAACCTCGCGCCTTACCTGTTGAGTAATAGTCAAAAGCCTCCGGTCGGAGGCTTTTGACTTTCTGCT
TACTGAATTTCGGTGTTAagcaccggtggagtgacgaccttcagcacgttcgtactgttcaacgatggtgtagtc
ttcgttgtgggaggtgatgtccagtttgatgtcggttttgtaagcacccggcagctgaaccggtttttagccat
gtaggtggttttaacttcagcgtcgtagtgaccaccgtctttcagtttcagacgcattttgatttcacctttcag
agcaccgtcttccgggtacatacgttcggtggaagcttcccaacccatggtttttttctgcataaccggaccgtc
ggacgggaagttggtaccacgcagtttaactttgtagatgaactcaccgtcttgcagggaggagtcctgggtaac
ggtaacaacaccaccgtcttcgaagttcataacacgttcccatttgaaaccttccgggaaggacagtttcaggta
gtccgggatgtcagccgggtgtttaacgtaagctttggaaccgtactggaactgcggggacaggatgtcccaagc
gaacggcagcggaccacctttggtaactttcagtttagcggtctgggtaccttcgtacggacgaccttcaccttc
accttcgatttcgaactcgtgaccgttaacggaaccttccatacgaactttgaaacgcatgaactctttgataac
gtcttcggaggaagcactagtCATTCATAATTTCTCCTCTTTAACGGGAGGATTGAAGAAGTGAAATTATGCGAG
GGCAACCATTATCACCGCCAGAGGTAAAATAGTCAACACGCACGGTGTTAattcgcggcctgcatAATTGTGAGC
GGATAACAATTGACATTGTGAGCGGATAACAAGATACTGAGCACGGGAGGGTTGATTGTGTGATTATGCGAGGCA
TCCTTAGCGAAAGCTAAGGATTTTTTTTtctagaTCCCTATCAGTGATAGAGATTGACATCCCTATCAGTGATAG
AGATACTGAGCACGGGAGGGTTGATTGTGTGAGTCTGTCACAGTTCAGCGGAAACGTTGATGCTGTGACAGATTT
ATGCGAGGCGGCTCCTTTTGGAGCCTTTTTTTTTagatctctgcagGCGAAAAACCCCGCCGAAGCGGGGTTTT
TTGCGGCCCTGCCTAGTCCTACCCTTCTTTGAGTGAGAACTAGGATCGCTCTCTCCTCCAGTGCTCAGTATCTCT
ATCACTGATAGGGATGTCAAACTCTATCAATGATAGAGTctcgagctgaccATAAATGTGAGCGGATAACATTGA
CATTGTGAGCGGATAACAAGATACTGAGCACCCCTGCCTAGTATCTCTTCTTTGCTTCCTCCAGTAAAGAGGAGA
TATTGGTTATGgggcccTCAACCAAGAAAAAGCCACTTACGCAGGAGCAACTGGAAGATGCTCGTCGTTTGAAAG
CCATCTACGAGAAAAAGAAAAATGAACTTGGCTTATCCCAAGAGTCTGTAGCGGATAAAATGGGTATGGGGCAGA
GTGGAGTGGGCGCGCTTTTTAACGGTATTAATGCTTTAAACGCATACAATGCAGCATTATTAGCAAAAATCCTGA
AAGTAAGTGTTGAAGAGTTCAGTCCTTCCATTGCTCGTGAAATCTACGAGATGTATGAGGCGGTTTCTATGCAAC
CGTCCTTACGTAGCGAGTATGAATACCCGGTGTTCTCCCACGTTCAAGCGGGCATGTTTAGTCCCGAACTTCGTA
CGTTTACAAAGGGCGACGCGGAGCGTTGGGTGAGTACCACGAAAAAGGCATCTGACTCTGCTTTTTGGCTGGAGG
TAGAAGGTAACTCCATGACGGCACCCACTGGAAGTAAACCCTCATTCCCGGATGGGATGCTGATTTTGGTTGACC
CCGAACAGGCTGTAGAACCAGGGGATTTTTGCATCGCCCGTCTTGGAGGCGATGAGTTTACCTTTAAGAAACTTA
TCCGTGACAGTGGACAAGTTTTTTTGCAACCGCTTAATCCGCAGTATCCCATGATTCCGTGCAATGAATCCTGTA
GCGTGGTGGGGAAAGTAATTGCGAGCCAGTGGCCCGAAGAAACATTCGGTTAACAAAGCCCGCCGAAAGGCGGGC
TTTTCTGTggatcc

Table listing our plasmids

Plasmid	Function	Genetics
pRAJ11	AND gate	GFPmut3b, RAJ11, pUC ori, ampR
pRHA12	AND gate	sfGFP, RAJ12, pSC101m ori, kanR
pRHA25	OR gate	mRFP1, RAJ11, pUC ori, ampR
pRHA37	OR gate	mRFP1, RAJ11, pUC ori, ampR
pRHA36	XOR gate (failed)	mRFP1, RAJ11, cI, RR12, pUC ori, ampR
pRHA40	XOR gate	mRFP1, RAJ11, cI, RAJ21, pUC ori, ampR

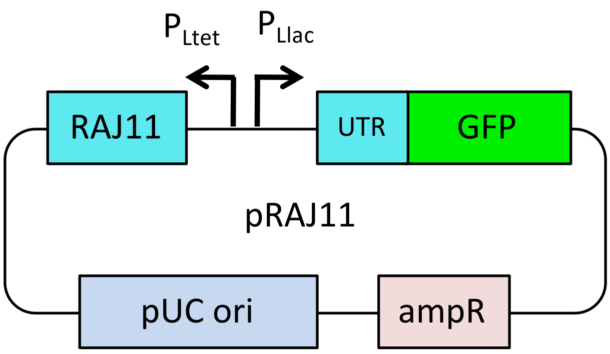

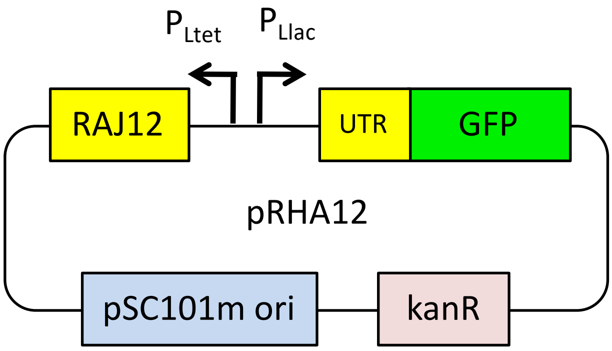

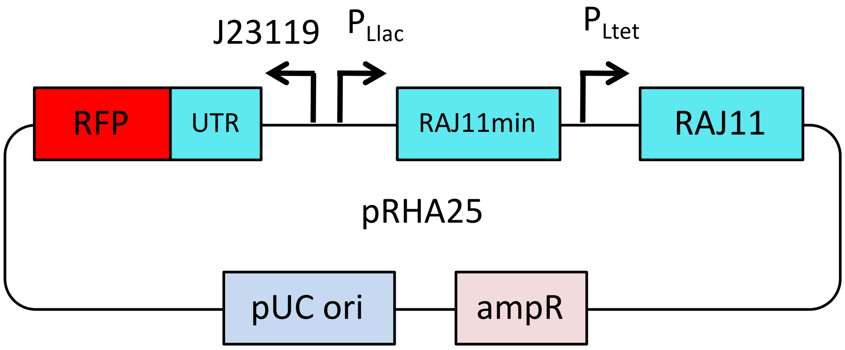

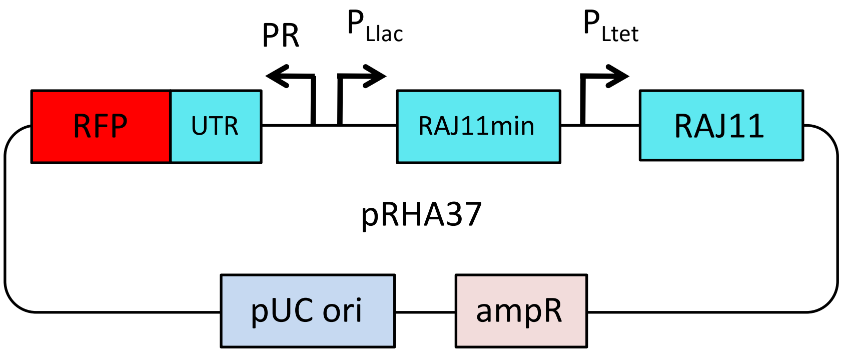
PR
P_{Llac}
P_{Ltet}
RFP
UTR
RAJ11min
RAJ11
pRHA37
pUC ori
ampR

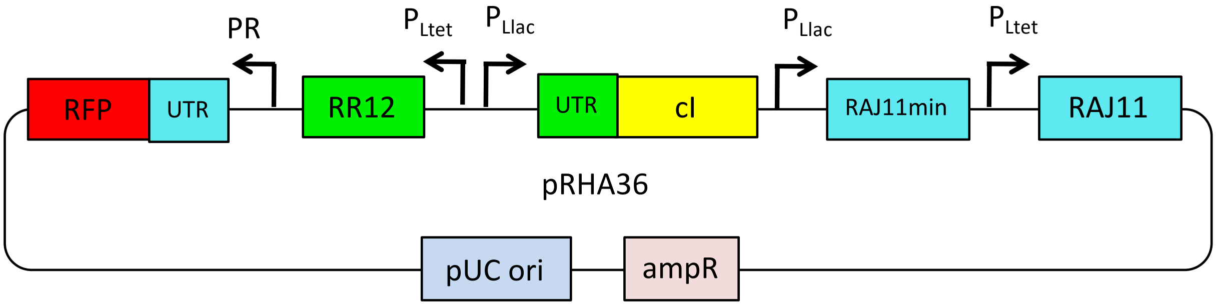
PR
P_{Ltet}
P_{Llac}
P_{Llac}
P_{Ltet}
RFP
UTR
RR12
UTR
cI
RAJ11min
RAJ11
pRHA36
pUC ori
ampR

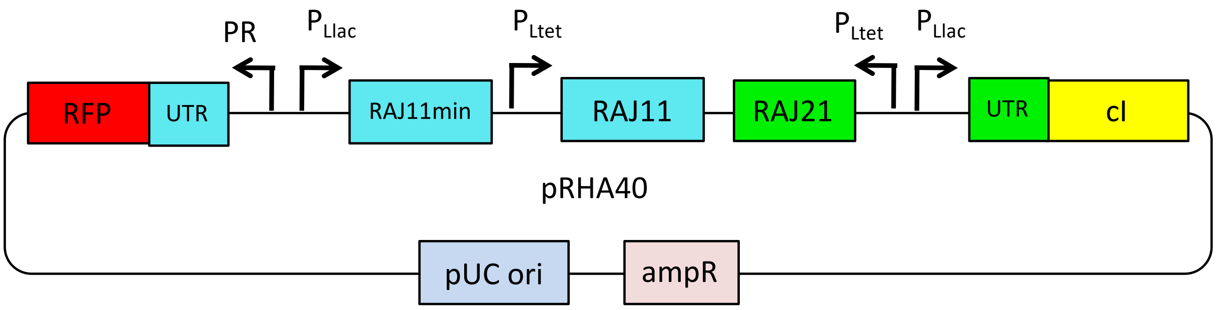
PR
P_{Llac}
P_{Ltet}
P_{Ltet}
P_{Llac}
RFP
UTR
RAJ11min
RAJ11
RAJ21
UTR
cI
pRHA40
pUC ori
ampR
